# Clinical pharmacology and pharmacogenetics of prostaglandin analogues in glaucoma

**DOI:** 10.3389/fphar.2022.1015338

**Published:** 2022-10-12

**Authors:** Lin Zhou, Wenyi Zhan, Xin Wei

**Affiliations:** ^1^ Department of Ophthalmology, West China Hospital, Sichuan University, Chengdu, China; ^2^ West China School of Medicine, Sichuan University, Chengdu, China

**Keywords:** glaucoma, prostaglandin analogues, efficiency, safety, pharmacogenetics, visual loss, neuropathy

## Abstract

Glaucoma is the main cause of irreversible visual loss worldwide, and comprises a group of progressive, age-related, and chronic optic neuropathies. Prostaglandin analogs are considered a first-line treatment in the management of glaucoma and have the best efficacy in reducing intraocular pressure. When comparing these therapeutic agents between them, long-term therapy with 0.03% bimatoprost is the most effective followed by treatment with 0.005% latanoprost and 0.004% travoprost. The prevalence of adverse events is lower for latanoprost than for other prostaglandin analogs. However, some patients do not respond to the treatment with prostaglandin analogs (non-responders). Intraocular pressure-lowering efficacy differs significantly between individuals partly owing to genetic factors. Rs1045642 in *ABCB1*, rs4241366 in *SLCO2A1*, rs9503012 in *GMDS*, rs10306114 in *PTGS1*, rs11568658 in *MRP4*, rs10786455 and rs6686438 in *PTGFR* were reported to be positive with the response to prostaglandin analogs in patients with glaucoma. A negative association was found between single nucleotide polymorphisms of *PTGFR* (rs11578155 and rs6672484) and the response to prostaglandin analogs in patients with glaucoma. The current review is an analysis of the information relevant to prostaglandin analog treatments based on previous literatures. It describes in detail the clinical pharmacology and pharmacogenetics of drugs belonging to this therapeutical class to provide a sound pharmacological basis for their proper use in ophthalmological clinical practice.

## Introduction

Glaucoma is the second leading cause of blindness worldwide and comprises a group of irreversible, progressive, and chronic optic neuropathies that result in vision loss owing to the death of retinal ganglion cells (Quigley and Broman, 2006; Kang and Tanna, 2021). Age is considered a major risk factor for glaucoma (McMonnies, 2017) and the prevalence of this pathology increases with age. Reports indicate a prevalence of 2.93% among patients aged 40–80 years and 10.0% among those over the age of 90 (Schuster et al., 2020). Although the exact mechanism is unknown, several factors seem to contribute. Firstly, increasing age may affect neuronal function, making older patients more susceptible to glaucoma. Additionally, fewer neurons may be detected, allowing for earlier identification of progressive changes in the optic nerve (Park et al., 2016).

Glaucoma is classified into open-angle glaucomas (OAGs), angle-closure glaucoma (ACGs) based on the anatomic status of the anterior chamber angle (Kang and Tanna, 2021). The most frequent type may differ from one region of the world to another (Schuster et al., 2020; Camara et al., 2022). For instance, ACGs is more prevalent in certain regions of Asia, whereas OAGs is more equally distributed throughout the world. Worldwide, the latter is the most frequent form of the disease.

To date, the precise factors that trigger the cascade of cellular events leading to glaucoma are poorly understood. However, this pathology is associated with an interplay between several risk factors, such as elevated intraocular pressure (IOP), older age, increased cup-to-disc ratio, thinner central cornea, and family history (Angeli and Supuran, 2019; Senthil et al., 2021). There are no available treatments for reversing the damage that glaucoma inflicts on the visual system. Early diagnosis and prompt treatment are important to prevent progression of vision loss. Increased intraocular pressure (IOP) is thought to damage the lamina cribrosa and is considered a major risk factor for glaucoma (Da Silva and Lira, 2022). Elevated IOP currently represents the only modifiable risk factor targeted by therapy for the prevention of glaucoma progression (Schuster et al., 2020; Zukerman et al., 2021). IOP is dependent on the balance of aqueous humor production, outflow of aqueous humor, and pressure of the episcleral vein (Islam and Spry, 2020). Aqueous humor is produced by the ciliary body, and there are two pathways for its outflow: a conventional pathway (the trabecular meshwork and Schlemm canal) and an unconventional one (uveoscleral outflow pathway) (Fautsch and Johnson, 2006; Goel et al., 2010). In the conventional pathway, aqueous humor passes through the trabecular meshwork into Schlemm’s Canal, then moves into an intrascleral venous plexus, and eventually to aqueous and episcleral veins (Costagliola et al., 2020). In the unconventional pathway, aqueous humor moves through the ciliary body of the angle, into ciliary body clefts, draining either into the supraciliary space, through the sclera, or into lymphatics (Yucel et al., 2009).

Topical and systemic medications, laser treatments, and surgical procedures are currently used to lower intraocular pressure (Kang and Tanna, 2021; Meier-Gibbons and Toteberg-Harms, 2021). Prostaglandin analogs (PGAs) are considered as a front-line medications for the treatment of glaucoma owing to their clinical efficacy to reduce IOP, mode of administration (once-daily dosing), and minimal side-effect profile (Tang et al., 2019; Aihara, 2021; Casson, 2022).

## Physiology of prostaglandins and implications for the treatment of glaucoma

Prostaglandins are pro-inflammatory molecules produced when arachidonic acid is metabolized by cyclooxygenase enzymes (isoforms COX-1 and COX-2). Basal levels of prostaglandins are produced by COX-1, and further increased by COX-2 isoform (Korbecki et al., 2014). There are five classes of prostaglandins: prostaglandin E2 (PGE2), F2 (PGF2), I2 (PGI2), D2 (PGD2), and thromboxane A (TXA2). Prostaglandin H2 (PGH2), a by-product of arachidonic acid metabolism by COX, is converted to each PH species by various synthases (Winkler and Fautsch, 2014; Angeli and Supuran, 2019; Aihara, 2021). Different prostaglandins interact with their corresponding G-protein-coupled receptors (GPCR) to elicit different responses. To date, nine such specific GPCRs are known to be expressed on different cell surfaces: DP1 and 2 receptors (DPs) for PGD2, EP1, 2, 3, and 4 receptors for PGE2, FP receptor for PGF2, and TP receptors for TXA2 (Aihara, 2021).

In human beings, the expression of FP receptor protein had been detected in the corneal epithelium, ciliary epithelium, the circular portion of ciliary muscle, and iris stromal and smooth muscle cells (Davis and Sharif, 1999; Sharif et al., 1999; Schlotzer-Schrehardt et al., 2002; Zhang and Yin, 2002). FP receptors activate phosphatidylinositol metabolism through G-coupled proteins, resulting in the increase of intracellular free calcium concentrations and modulation of various signaling cascades. PGF2α and prostaglandin FP agonists decrease IOP by increasing uveoscleral outflow *via* an unconventional pathway. PGAs could activate prostaglandin receptors in ciliary muscle, iris root and sclera (Park et al., 2021). The possible mechanism of PGAs including the relaxation of ciliary smooth muscles, the alteration of cytoskeletal, remodeling of the extracellular matrix of the uveoscleral pathway (Tripathy and Geetha, 2022). In addition, it might also enhance some aqueous fluid outflow *via* the FP-receptors present in the trabecular meshwork (Sharif et al., 2003). However, the underlying mechanisms of this effect are not fully understood (Lindsey et al., 1997). FDA approved latanoprost, the first FP-related drug for the treatment of glaucoma, in 1996. Because of its high efficiency, it has been the first-line drug against this pathology. Subsequently, similar FP agonists (bimatoprost, travoprost, and tafuprost) were approved.

The EP receptor is expressed in the trabecular meshwork and ciliary body. The activation of EP2/EP4 receptors results in decreased cell stiffness in Schlemm’s canal, increased cell contractility of the trabecular meshwork, and decreased IOP *via* the conventional outflow pathway (Wan et al., 2007; Wang et al., 2013). Previous animal studies have also reported that EP2 and EP4 agonists decrease IOP (Crider and Sharif, 2001; Saeki et al., 2009; Boussommier-Calleja et al., 2012; Bertrand et al., 2021). In 2018, a novel EP2 receptor agonist, omidenepag isopropyl (OMDI), was shown to reduce IOP by increasing both uveoscleral and trabecular outflow in animals. Later, it was proven to also be effective in humans (Aihara et al., 2020; Shiratori et al., 2021).

PGD2 has been reported to play important roles in reproduction, allergic inflammation, immune response, and sleep regulation. For the ocular system, it is involved in the immune responses (Jung et al., 2014; Doucette and Walter, 2017). PGD2 has been proved to increase the production of aqueous humour, but it also shows an increase amount of uveoscleral outflow *via* an unconventional pathway (Sharif et al., 2004). PGI2 has been considered as an effective vasodilator and an anti-platelet aggregation agent (Hara et al., 2005). It also plays important a role in the preventing apoptosis through PPAR pathway (Nana-Sinkam et al., 2007). In addition, it has been proven to reduce IOP in rabbits and beagles in previous study (Hoyng and de Jong, 1987). TXA2 is shown to promote the activation of the platelets, induce bronchodilation, and promote the proliferation of respiratory smooth muscle cells (Christman et al., 1992; Rolin et al., 2006). In 2013, TPs had been shown to induce high concentration contraction of porcine ciliary vessels *in vitro* (Kringelholt et al., 2013). However, no prostaglandin analogues (PGD2, PGI2, and TXA2) available for reducing IOP are reported up to now.

## Clinical efficacy of prostaglandin analogues

**FIGURE 1:**
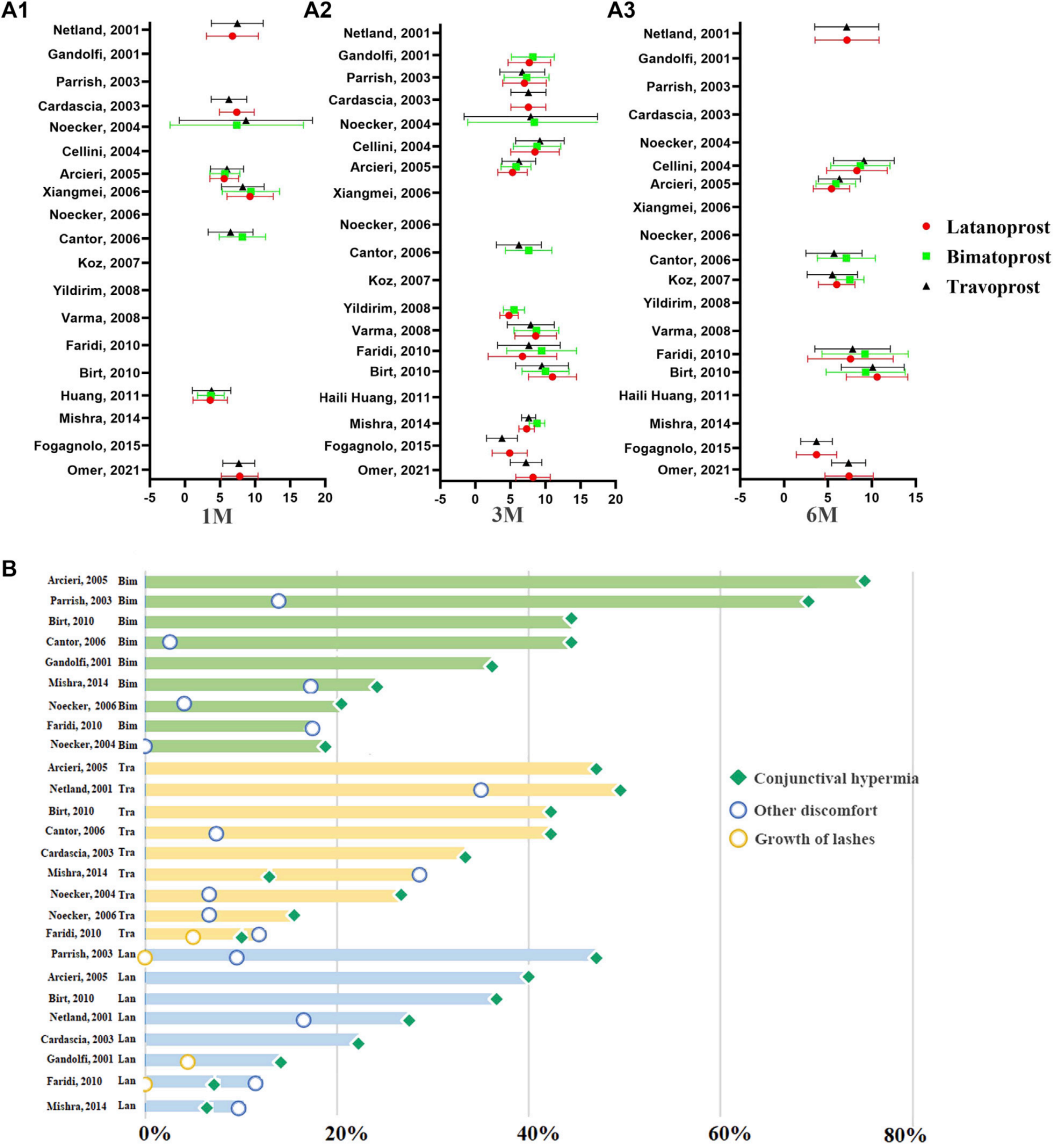
The distribution of IOP-lowering efficacy of PGAs at different time points of therapy and the distribution of ocular side events of PGAs in previous RCTs. **(A)** The distribution of the IOP-lowering efficacy of PGAs at 1M, 3M, and 6M. The abscissa represents the mean IOP reduction from baseline to the endpoint. The ordinate represents the data from different reported literatures. **(A1)**: The distribution of IOP-lowering efficacy of PGAs at 1 month. **(A2)**: The distribution of IOP-lowering efficacy of PGAs at 3 months. **(A3)**: The distribution of IOP-lowering efficacy of PGAs at 6 months. Red circle: Latanoprost; Green square: Bimatoprost; Dark triangle: Travoprost. **(B)** The distribution of ocular side events of PGAs in previous RCTs. The horizontal axis represents the incidence of ocular side effects. The pale green columns in the top represent the ocular side effects in Bimatoprost. The faint yellow columns in the middle represent the ocular side effects in Travoprost. The pale blue columns in the bottom represent the ocular side effects in Latanoprost. Green square: conjunctival hyperemia; yellow circle: growth of eyelashes; blue circle: other discomfort.

PGAs (bimatoprost, latanoprost, travoprost, tafuprost and omidenepag isopropyl) are the most efficacious drugs in controlling IOP, followed by β-blockers, α-2 agonists, and carbonic anhydrase inhibitors (Li et al., 2016; Inoue et al., 2020). Several clinical trials have compared the efficacy and side effects of various PGAs. However, the results of these studies are inconsistent. For example, Florent Aptel demonstrated that 0.03% bimatoprost is more effective in reducing IOP than 0.005% latanoprost and 0.004% travoprost (Aptel et al., 2008). Conversely, Denis (Denis et al., 2007) reported that 0.004% travoprost and 03% bimatoprost might have a greater efficacy in reducing IOP than 0.005% latanoprost. Additionally, these studies were undertaken years ago. To provide a summary of the efficacy and adverse events of different PGAs, we selected relevant randomized controlled studies published between 1 January 2000, and 1 April 2022. The keywords were “glaucoma,” “ocular hypertension,” “prostaglandin analogues” “latanoprost,” “travoprost,” “bimatoprost,” “tafuprost,” “omidenepag isopropyl,” “intraocular pressure,” “randomized controlled trial.” Reports on the comparative efficacy of tafuprost, omidenepag isopropyl and other newer PGAs were scarce. We did not analyze the efficacy and side effects of tafuprost, omidenepag isopropyl. Finally, 19 studies were included in this analysis (Figure 1) (Gandolfi et al., 2001; Netland et al., 2001; Cardascia et al., 2003; Noecker et al., 2003; Parrish et al., 2003; Cellini et al., 2004; Arcieri et al., 2005; Cantor et al., 2006; Noecker et al., 2006; Koz et al., 2007; Varma et al., 2008; Yildirim et al., 2008; Birt et al., 2010; Faridi et al., 2010; Huang et al., 2011; Mishra et al., 2014; Fogagnolo et al., 2015; Muz et al., 2021).

To accurately compare the efficacy and side effects of these therapeutical agents, we undertook a subgroup analysis based on the duration of drug administration. The result was expressed as the absolute change in mmHg from baseline to the endpoint of the treatment. No significant difference in the IOP reduction was observed following 1 month of treatment with 0.03% bimatoprost (7.49 ± 4.57 mmHg, *n* = 155), 0.005% latanoprost (7.00 ± 3.64 mmHg, *n* = 311) or 0.004% travoprost (7.07 ± 3.87 mmHg, *n* = 387). Additionally, there was no significant difference in IOP reduction after one, three, and 6 months of treatment with 0.005% latanoprost and 0.004% travoprost. While three and 6 months of therapy with 0.03% bimatoprost are more effective for IOP control than three and 6 months of therapy with 0.004% travoprost or 0.005% latanoprost (0.03% bimatoprost: 8.13 ± 3.59, 7.93 ± 3.81; 0.004% travoprost: 7.21 ± 3.64, 6.95 ± 3.64; 0.005% latanoprost: 7.63 ± 3.29, 7.25 ± 3.80 respectively).

## Systemic and ocular adverse effects of anti-glaucoma prostaglandin analogues

Similar to other medications, drugs against glaucoma have specific adverse effects (Arbabi et al., 2022). However, prostaglandin analogs administered for the treatment of glaucoma have an optimal safety profile in terms of systemic adverse events, and some of the side effects are only of cosmetic significance (Wang et al., 2021). These agents are safe for the cardiovascular and respiratory systems and do not induce any related-adverse events (Waldock et al., 2000; Hollo, 2007; Alm et al., 2008). Headache is a potential systemic adverse event of PGAs therapy (Wang et al., 2021; Arbabi et al., 2022), these agents may induce headaches or activate migraines in some individuals (Antonova et al., 2013). Headaches are reversible after the cessation of PGAs treatment (Wang et al., 2021; Arbabi et al., 2022).

Ocular side effects include conjunctival hyperemia, growth of eyelashes, and other discomforts (eye irritation, itching, tearing, foreign body sensation, iris cysts, cystoid macular edema, anterior uveitis, and reactivation of herpes simplex keratitis) (Hollo, 2007; Alm et al., 2008; Silvio et al., 2018). Conjunctival hyperemia is the most frequent mild transient ocular adverse effect in patients receiving PGAs. It seems to be caused exclusively by vasodilation because associated-inflammation has not been previously documented (Lee et al., 2019). Additionally, severe hyperemia is associated more frequently with PGAs therapy than with other anti-glaucoma drugs (Li et al., 2018). However, the severity of conjunctival hyperemia tends to decrease as the treatment continues (Sakata et al., 2016).

The growth of lashes (darker and longer eyelashes) is an interesting and well-documented side effect associated with the use of PGAs. In a retrospective study in 1997, Johnstone reported 43 individuals receiving topical PGAs therapy suffered hypertrichosis and growth of lashes (Johnstone, 1997). Changes related to eyelashes include their number, thickness, length, and curvature (Johnstone, 1997). A total of 46.3% of individuals with adaptation to PGAs presented changes in the thickness and length of the eyelashes (Chiba et al., 2004). The average length of eyelashes at baseline and following sixth months of latanoprost therapy was 5.5–6.1 mm and 6.2–6.8 mm, respectively (Elgin et al., 2006). Additionally, bimatoprost gel suspension applied on the eyelashes for 6 weeks resulted in a statistically significant growth of eyelashes length (average length: 2.0 mm) (Wester et al., 2010).

We summarized the ocular adverse effects presented in the 11 RCT studies comprised in this analysis. These included conjunctival hyperemia, growth of lashes, and other discomfort associated with the topical administration of latanoprost, bimatoprost, and travoprost (Gandolfi et al., 2001; Netland et al., 2001; Cardascia et al., 2003; Noecker et al., 2003; Parrish et al., 2003; Arcieri et al., 2005; Cantor et al., 2006; Noecker et al., 2006; Birt et al., 2010; Faridi et al., 2010; Mishra et al., 2014) (Figure 1). Conjunctival hyperemia was the most frequent ocular side effect of all three prostaglandin analogs. The prevalence of conjunctival hyperemia in travoprost (42.23%) and bimatoprost (43.57%) groups was significantly higher than in latanoprost (27.62%) group (*p* = 1.22E-8). Patients who received travoprost had a higher prevalence of eyelash growth than those receiving latanoprost and bimatoprost (*p* = 5.16E-4). In summary, latanoprost therapy has the lowest prevalence of adverse events among these PGAs.

## Pharmacogenetics and prostaglandin analogues

Pharmacogenetics is the field of research on the contribution of genetic factors to drug treatment outcomes. Interindividual genetic variations can affect the bioavailability, efficacy, metabolism, and toxicity of medicines. The primary goal is to help pharmacologists develop more effective and safer drugs by considering genetic factors (Fini et al., 2017; Relling et al., 2020). Detailed knowledge of drug pharmacogenetics enables the optimization of therapy by dosage customization according to the patient’s genetic profile. Though “pharmacogenetics” was raised by Friedrich Vogel in 1959, the relevance of inherited genetic traits in affecting clinical outcomes had been detected long before (Daly, 2017; Fini et al., 2017). Pharmacogenetics has been successfully used for developing treatments of several diseases, such as PXT3003 for Charcot-Marie-Tooth (Attarian et al., 2014; Mandel et al., 2015) and Parkinson’s disease (Hajj et al., 2015), inhibitors of the epidermal growth factor receptor and anaplastic lymphoma kinase, for the treatment of various types of cancer (Kerr et al., 2014; Morganti et al., 2019). Single nucleotide polymorphisms (SNPs) and small indels are the most frequent and extensively studied genetic variations that affect pharmacokinetics, efficacy, and toxicity of drugs. Other studies indicated that genomic structural variations, such as inversions, translocations, and copy number variants (CNVs), are also a rich source of genetic variability (Gao et al., 2015).

To date, 65 SNPs in 47 genes are reported to be associated with POAG, 13 SNPs in 19 genes with PACG, and 11 SNPs in 18 genes with pseudoexfoliation (Zukerman et al., 2020). Linkage, genome-wide association, and candidate gene studies have identified several loci (TMCO1, CDKN2B-AS1, CAV1, and CAV2, AFAP1 GMDS, et al.) responsible for the development of glaucoma.

**FIGURE 2:**
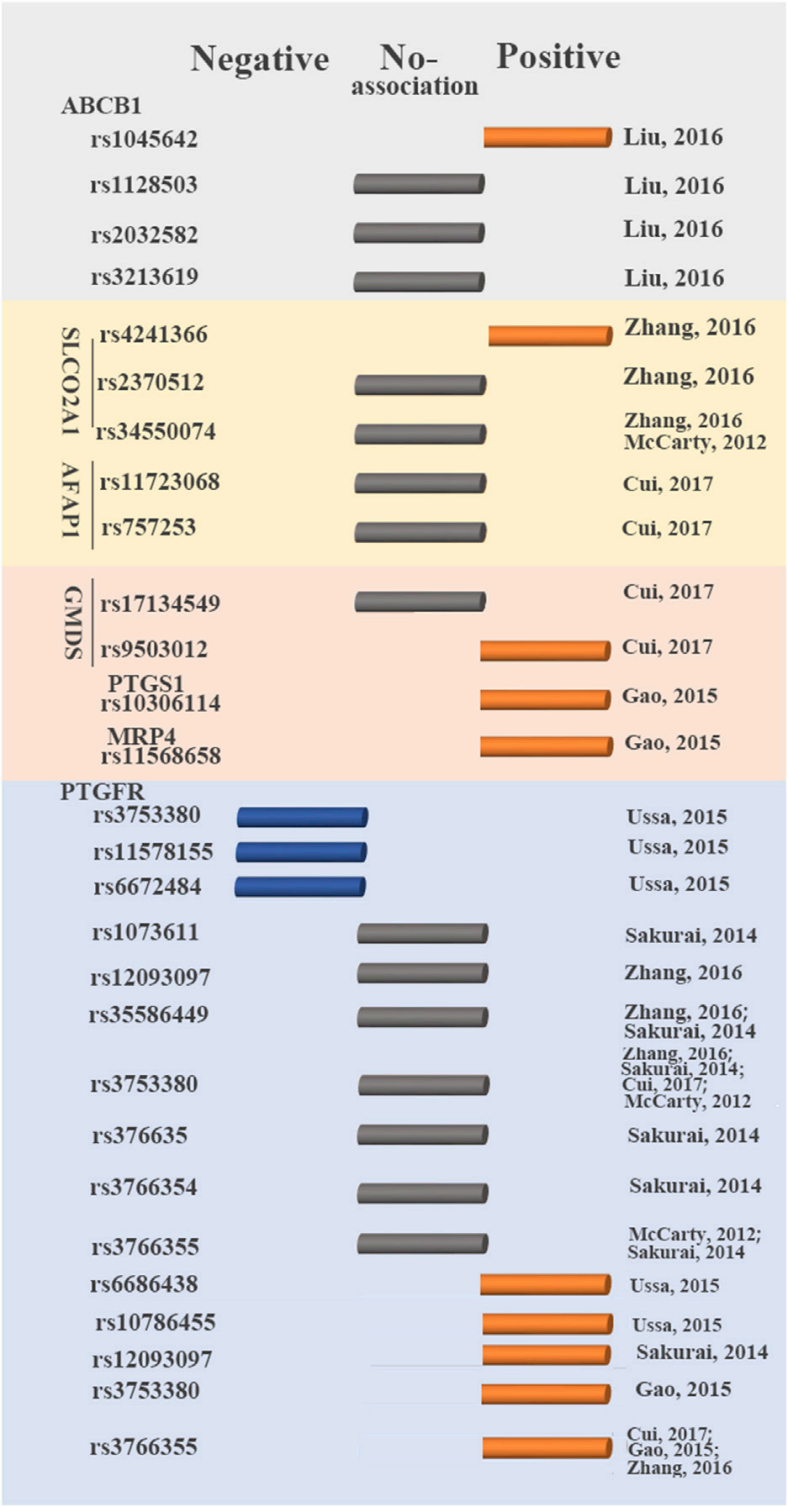
Genetic variants that influence latanoprost therapy outcomes. Blue circular cylinder: negative association; gray circular cylinder: no association; orange circular cylinder: positive association.

The efficacy of anti-glaucoma drugs significantly differs between patients with glaucoma, and some anti-glaucoma drugs may prove ineffective in some individuals with glaucoma (Inoue et al., 2016). To date, the response to latanoprost of patients with glaucoma and ocular hypertension, is reported to be associated with the genetic polymorphism of seven genes (ABCB1, SLCO2A1, AFAP1, GMDS, PTGS1, MRP4, and PTGFR) (McCarty et al., 2012; Sakurai et al., 2014; Gao et al., 2015; Ussa et al., 2015; Liu et al., 2016; Zhang et al., 2016; Cui et al., 2017) (Figure 2). However, such studies on pharmacogenetics have not yet been conducted for other PGAs.

Prostaglandin F2 receptor negative regulator (PTGFR) is a primary target for latanoprost. Latanoprost activates PTGFR to produce Gq-mediated inositol triphosphate-3, increasing the levels of intracellular calcium and matrix metalloproteinases (MMP) (Hinz et al., 2005). Increasing the activity of MMP reduces the extracellular matrix in ciliated muscle, increases the outflow of aqueous humor and consequently reduces IOP (Zhang et al., 2016). Up to now, six studies reported the association between the SNPs in PTGFR (rs1073611, rs10786455, rs11578155, rs12093097, rs35586449, rs3753380, rs376635, rs3766354, rs3766355, rs6672484, and rs6686438) and glaucoma with the treatment of latanoprost (McCarty et al., 2012; Sakurai et al., 2014; Gao et al., 2015; Ussa et al., 2015; Zhang et al., 2016; Cui et al., 2017). Among these SNPs, four were not correlated with the response to latanoprost (rs1073611, rs35586449, rs376635, rs3766354). SNPs (rs10786455, rs6686438) were associated with a positive response to latanoprost. Conversely, rs11578155 and rs6672484 were associated with a negative response to latanoprost. These studies on the association between SNPs (rs12093097, rs3753380, rs3766355) in PTGFR and the change in IOP to prostaglandin analogs showed inconsistent in different studies (Sakurai et al., 2014; Gao et al., 2015; Ussa et al., 2015; Zhang et al., 2016).

The correlation between the remaining 13 SNPs in six genes and IOP-lowering efficacy of latanoprost in patients with glaucoma was also investigated (McCarty et al., 2012; Gao et al., 2015; Zhang et al., 2016). SLCO2A1 is a transporter of latanoprost acid highly expressed in human ocular tissues (Fuchsjager-Mayrl et al., 2005; Zhang et al., 2016). Actin filament-associated protein (AFAP) can affect the actin filament and cytoskeleton and plays important roles in aqueous outflow (Fuchsjager-Mayrl et al., 2005; Cui et al., 2017). GDP-mannose 4,6-dehydratase (GMDS) is required for the first step of *de novo* fucose synthesis (Gharahkhani et al., 2014). Fucose is necessary for various biological functions, including growth factor receptor signaling. It had been reported to corrected with the development of glaucoma (Becker and Lowe, 2003; Miyoshi et al., 2008). Multidrug resistance protein 4 (MRP4) is expressed in human trabecular meshwork cells and thus, in the human aqueous humor outflow pathway (Pattabiraman et al., 2013; Gao et al., 2015). Prostaglandin-endoperoxide synthase 1 (PTGS1/COX-1) plays important roles in the production of prostaglandins. Rs1128503, rs2032582 and rs3213619 in ABCB1, rs2370512 in SLCO2A1, rs34550074 in SLCO2A1, rs11723068 and rs757253 in AFAP1, rs17134549 in GMDS showed no association with the response to latanoprost (Gao et al., 2015; Liu et al., 2016; Zhang et al., 2016; Cui et al., 2017). Rs1045642 in ABCB1, rs11568658 in MRP4, rs4241366 in SLCO2A1, rs9503012 in GMDS and rs10306114 in PTGS1 were correlated with positive responses to latanoprost (Gao et al., 2015; Zhang et al., 2016; Cui et al., 2017).

Different effects of the same SNPs on the efficacy of latanoprost might be caused by different ethnicities, different diagnostic criteria for glaucoma and the minor error between prostaglandin analogs and latanoprost (Canut et al., 2021). The frequencies of the different variants showed prominent interethnic variability. Therefore, this might be a possible and important factor in the response to PGAs in the treatment of glaucoma.

## Discussion

In summary, we investigated the clinical pharmacology and pharmacogenetics of PGAs in glaucoma patients. Long-duration treatment with 0.03% bimatoprost is more effective than those with 0.005% latanoprost and 0.004% travoprost. Latanoprost shows the lowest prevalence of adverse events among the other PGAs. Data on the recently developed EP2 receptor agonist OMDI are limited. The IOP-lowering efficacy of OMDI was noninferior to latanoprost and may also be used as a first-line drug to treat glaucoma (Aihara et al., 2020).

More studies are needed to demonstrate its efficacy, identify the associated-adverse effects and study on the pharmacogenetics of PGAs. Individuals and ethnic populations with different genetic background may show significant differences in drug metabolism and efficacy, sometimes manifesting as severe adverse drug reactions or lack of efficacy. Ophthalmologists need to understand the clinical pharmacology and pharmacogenetics of PGAs and prescribe them accordingly to maximize their beneficial effects.
